# Preclinical evaluation of a compliant and absorbable cardiac implantable electronic device antibacterial envelope

**DOI:** 10.1093/europace/euaf260

**Published:** 2025-10-10

**Authors:** Robert D Schaller, Suneet Mittal, Mauro Biffi, Matteo Ziacchi, Nicole Kirchhof, Aldo Ferrari, Alexander Breitenstein

**Affiliations:** Electrophysiology Section, Division of Cardiology, Hospital of the University of Pennsylvania, 3400 Spruce Street, Philadelphia, PA 19104, USA; Snyder Center for Comprehensive Atrial Fibrillation and Department of Cardiology, Valley Health System, Paramus, NJ, USA; Institute of Cardiology, IRCCS Azienda Ospedaliero Universitaria di Bologna, Via Massarenti 9, Bologna 40125, Italy; Institute of Cardiology, IRCCS Azienda Ospedaliero Universitaria di Bologna, Via Massarenti 9, Bologna 40125, Italy; Thinkpathology LLC, Minnetonka, MN, USA; Hylomorph AG, Zurich, Switzerland; Electrophysiology, Department of Cardiology, University Hospital Zurich, Zurich, Switzerland

**Keywords:** Cardiac implantable electronic device, CIED envelope, Pocket health, Pocket stability, Pocket infection, Antibiotic elution, Histopathology

## Introduction

The implantation of cardiac implantable electronic devices (CIEDs) is associated with potential complications, including infection and device migration. Infection rates are reported between 1.7 and 5.6%, tripling all-cause mortality.^1,2^ To reduce risk, absorbable antibacterial-eluting envelopes such as TYRX™ (Medtronic, Inc., Minneapolis, MN) are used in high-risk patients.^3^ Clinical trials have shown that TYRX significantly reduces the incidence of major CIED infections without increasing procedural complications.^4^

Envelope usability is essential for long-term device stability and minimizing adverse events. Vesta™ (Hylomorph AG, Zurich, CH) represents a novel CIED envelope, engineered to offer both antimicrobial protection and improved biomechanical properties. Its enhanced form-fitting design allows for closer conformity to the device and surrounding tissue, potentially reducing pocket volume and mechanical irritation, promoting healthier capsule formation, and facilitating future pocket access.

## Methods and results

Vesta comprises two distinct components. The absorbable mesh is constructed from polyglycolic acid (PGA) microfibers knitted into a seamless three-dimensional sleeve.^5^ The antibiotics are embedded in an absorbable PLGA-based film, fabricated via solvent casting. Each film contains 11.9 mg of rifampin and 7.6 mg of minocycline; the same dosage used in TYRX.

**Figure 1:**
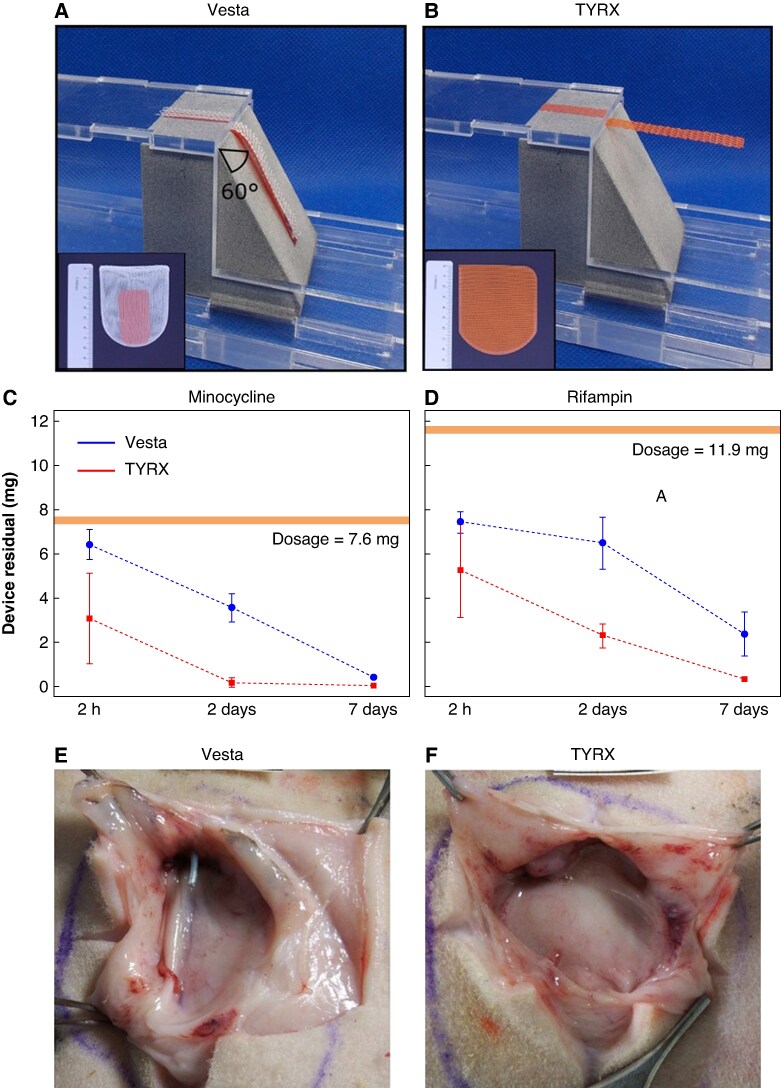
(*A–B*) Vesta conforms perfectly up to a curvature of 60°. (*C–D*) *In vivo* antibiotic residual in the Vesta (blue) and TYRX (red) in rabbit. Data points depict the mean residual amount of minocycline and rifampin in the devices. Error bars = standard deviation. (*E–F*) Widely opened Vesta and TYRX pockets in the subcutis of sheep immediately after device removal (Week 13; envelopes absorbed).

To evaluate the form-fitting behaviour of Vesta, the ISO 9073-7 standard was adopted.^6^ The dry Vesta mesh and drug-eluting film conformed to non-planar surfaces with inclination up to 60°. Upon wetting, drapability improved to conform to the steepest tested inclination (75°). The TYRX mesh failed to conform at the lowest inclination of 15° in both the dry and wet configurations (*Figure* *1A* and *B*).

The antimicrobial efficacy of Vesta against multiple bacterial strains (methicillin-resistant Staphylococcus aureus, *Staphylococcus epidermidis*, *Enterococcus faecium*, *Escherichia coli*, *Pseudomonas aeruginosa*, and *Acinetobacter baumannii*)^7^ was evaluated *in vitro* using a bacterial challenge assay adapted from AATCC TM100 (2019). Vesta demonstrated a statistically significant 6- to 7-log reduction in bacterial counts. No viable bacteria were recovered.

A pharmacokinetics (PK) study was conducted *in vivo*. Ten New Zealand White rabbits were included, each receiving two bilateral subcutaneous implants of non-functioning CIEDs with either Vesta (*n* = 11) or TYRX (*n* = 9). Animals were divided into three groups, euthanized at 2 hours, 2 days, and 7 days, respectively. Serum samples were collected at baseline, 2 and 8 hours, 1 day, and 1 week. Serum concentrations were low for both envelopes, with peak levels observed between 8 and 24 hours (129 ng/mL for minocycline and 151 ng/mL for rifampin). By 7 days post-implantation, serum concentrations of both antibiotics declined to the assay’s limit of detection (100 ng/mL).

Antibiotic elution dynamics were assessed by quantifying residual drug content in the explanted envelopes. Vesta demonstrated sustained, linear elution: 20% of the initial dose of each antibiotic was released within 2 hours, ∼50% by Day 2, and 80–90% by Day 7. TYRX sustained a rapid burst release, with 60% of both antibiotics eluted within the first 2 hours. No residual minocycline was detectable beyond 2 days, while rifampin was completely eluted by Day 7 (*Figure* *1C* and *D*).

To assess Vesta’s stabilization performance, pocket tissue response, and degree of device absorption, three female sheep (weight range 57–65 kg) were implanted with seven subcutaneous devices. Each animal received three non-functional CIEDs with lead segments placed in separate Vesta envelopes, two corresponding CIEDs with lead segments in separate TYRX envelopes, one bare CIED with a lead segment, and one small reference coupon of non-absorbable high-density polyethylene (HDPE). Landmark tattoos were applied to the skin. The animals were euthanized at 2, 9, and 13 weeks. Device migration was assessed by measuring displacement from the centroid of the landmark tattoos. Device migration remained clinically irrelevant and well below 3 cm at all implant sites.^8^ The differences between groups were not statistically significant.

All CIED pockets were healed and devoid of gross pathological findings. In Vesta pockets, capsule thickness increased from 0.5 ± 0.1 mm (Week 2) to 1.3 ± 0.3 mm (Week 9) and then thinned to 1.1 ± 0.4 mm (Week 13). Capsule thickness in TYRX pockets increased from 0.8 ± 0.1 mm (Week 2) to 1.0 ± 0.1 mm (Week 9) and to 1.5 ± 0.5 mm (Week 13). Despite minor differences, the histopathological findings in both groups were comparable and consistent with a typical remodelling response around bio-absorbable implants given the animal model and study duration (*Figure* *1E* and *F*).

## Discussion

Vesta features enhanced compliance and optimal form-factor. This superior design allows for Vesta to conform to commercially available CIEDs. The film contains the same dosage of minocycline and rifampin as the TYRX envelope. *In vitro* bacterial challenge confirmed that Vesta effectively kills the most common pathogens responsible for CIED infections.^9^  *In vivo* testing demonstrated sustained local elution of both antibiotics for at least 7 days as opposed to TYRX, which showed no residual minocycline beyond 2 days and complete elution of rifampin by Day 7. Long-term implantation in sheep demonstrated that Vesta’s stabilization, tissue response, and absorption were comparable to the established benchmark set by TYRX. Despite architectural differences between Vesta and TYRX, *in vivo* absorption dynamics were comparable and consistent with their degradation profiles.^10^

## Conclusions and limitations

This novel antibiotic-eluting envelope compares favourably to the current standard in infection prevention, due to its engineering design and drug elution profile. Its enhanced draping properties may improve ease of use during implantation, offering practical advantages in clinical workflows. Histopathological examination was devoid of features indicative of adverse healing. These findings indicate that the innovative Vesta envelope can provide a meaningful contribution to infection prevention in CIED implantation procedures.

This is a pre-clinical study. It was performed based on the current standards in comparison with TYRX. Clinical validation of the Vesta will be obtained after regulatory approval and will require initial adoption and follow-up.

## Data Availability

The data underlying this article will be shared on reasonable request to the corresponding author.
